# The safety window of blood magnesium in pulmonary complications of non-pulmonary sepsis: A U-shaped risk and prognostic analysis based on MIMIC-IV

**DOI:** 10.1371/journal.pone.0351216

**Published:** 2026-06-15

**Authors:** Taotao Peng, Yu Li, Yukun Ren, Mi Yang, Zonghong Long, Dukun Zuo, Lu Huang, Huawei Liu, Zhenxin Duan, Hong Li

**Affiliations:** Department of Anesthesiology, Second Affiliated Hospital, Army Medical University (Third Military Medical University), Chongqing, China; Versiti Blood Research Institute, UNITED STATES OF AMERICA

## Abstract

Pulmonary complications in non-pulmonary sepsis (PC-NPS) are the leading cause of morbidity and mortality in the intensive care unit. Early prevention and monitoring are paramount since the prevention strategies remain limited yet. Magnesium, an essential electrolyte involved in inflammation and vascular regulation, may influence the development of such complications. This retrospective cohort study used data from the MIMIC-IV database to explore the relationship between baseline serum magnesium levels and PC-NPS among 4,836 patients with non-pulmonary sepsis. Survival analysis demonstrated that patients who developed PC-NPS had significantly higher 90-day mortality compared with those without lung injury. When stratified by baseline serum magnesium quartiles, patients in the highest quartile (>2.1 mg/dL) showed the poorest survival. Multivariable logistic regression confirmed that elevated magnesium was independently associated with increased risk of PC-NPS, and restricted cubic spline modeling revealed a U-shaped, nonlinear association between baseline magnesium concentration and PC-NPS risk. Inflection points at 1.26 and 1.91 mg/dL identified a range of relatively lower risk. These findings suggest that baseline serum magnesium levels exhibit a U-shaped relationship with the risk of PC-NPS. Evaluating these levels may aid in clinical prognostication and the exploration of underlying mechanisms.

## Introduction

Sepsis, a syndrome of dysregulated host response induced by systemic infection, has become one of the leading causes of death in intensive care units worldwide [1]. Among its complications, pulmonary complications in non-pulmonary sepsis(PC-NPS) are one of the most common and lethal, with an incidence ranging from 25% to 45% and a mortality rate as high as 30% to 40% [2, 3]. Despite continuous advances in critical care medicine and supportive therapies, clinical treatments for sepsis-associated PC-NPS remain limited and often ineffective, severely impacting patient outcomes. The primary pathological mechanisms underlying these pulmonary complications involve a vicious cycle of inflammatory storms, disruption of the capillary-alveolar endothelial barrier, coagulation abnormalities, and oxidative stress. Currently, there is a lack of specific biomarkers for early warning of alveolar epithelial cell death in sepsis risk, leading to delayed intervention and further exacerbating the disease burden [4, 5, 6, 7].

In recent years, the role of electrolyte disturbances in the progression of sepsis has garnered increasing attention. As the second most abundant intracellular cation in the human body, magnesium ions are indispensable for maintaining cellular homeostasis and regulating immune responses. Dysregulation of magnesium levels has been proven to be closely associated with the pathogenesis and prognosis of sepsis, indicating its potential critical role in this pathological process. Hypomagnesemia exacerbates the inflammatory cascade by activating the HMGB1/TLR4/NF-κB pathway [8], while also inhibiting P2X7 receptor-mediated calcium signaling abnormalities, aggravating pyroptosis and mitochondrial dysfunction [9]. Clinical evidence further confirms that blood magnesium levels are closely related to microcirculatory dysfunction, lactate clearance rate, and coagulopathy risk in septic patients, exhibiting a J-shaped dose-effect relationship—both hypomagnesemia (<1.6 mg/dL) and hypermagnesemia (>2.4 mg/dL) are associated with increased mortality [10, 11, 12, 13, 14]. Notably, ferroptosis, a key mechanism of alveolar epithelial cell death in septic lung injury, is regulated by magnesium ions through modulation of NLRP3 inflammasome activity and the SLC7A11/GPX4 pathway [15, 16, 17, 18]. These findings suggest that dynamic changes in blood magnesium may influence the occurrence and progression of PC-NPS through immune-metabolic reprogramming.

Existing studies have indicated that electrolyte disturbances may affect the prognosis of sepsis, but research on biomarkers for PC-NPS has paid insufficient attention to blood magnesium homeostasis. Its predictive efficacy, optimal cutoff values, and mechanistic explanations remain unclear. It is worth noting that septic patients often experience fluctuations in blood magnesium due to interventions such as renal replacement therapy and parenteral nutrition. Therefore, studies on baseline monitoring strategies and their spatiotemporal relationship with lung injury are urgently needed. Based on this, this study aims to investigate the dose-effect relationship between baseline blood magnesium levels and PC-NPS, to evaluate its predictive value, thereby providing evidence for personalized risk stratification and targeted magnesium supplementation strategies in sepsis.

## Materials and methods

### Study participants

This study utilized the MIMIC-IV electronic database (version 3.0), jointly established by the Massachusetts Institute of Technology (MIT) and Beth Israel Deaconess Medical Center (BIDMC), to include hospitalized patients diagnosed with sepsis. Sepsis was identified using International Classification of Diseases (ICD-9 and ICD-10) codes (code range: 0380, A40, A400, A401, A408, A409, 0382, A403, 03810, 03811, 03812, 03819, A410, A4101, A4102, A411, A412, 0223, A227, A267, A327, 03842, A4151, 03843, A4152, 03844, A4153, 0031, A021, 03841, A413, A5486, A4181, A427, 0383, A414, B377, 0545).

**Fig 1:**
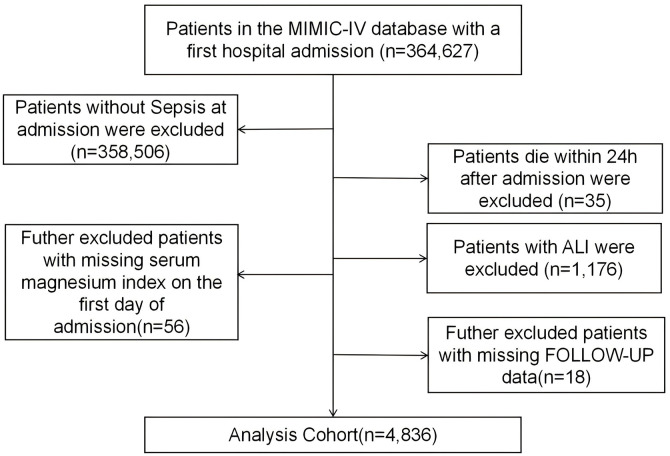
Flowchart for inclusion and exclusion of the Participants. This flowchart outlines the steps involved in selecting participants for the analysis cohort, starting from the initial dataset of 364,627 patients in the MIMIC-IV database. Patients were excluded based on various criteria, including the absence of sepsis at admission, death within 24 hours of admission, missing serum magnesium index, and missing follow-up data. The final analysis cohort consists of 4,836 participants.

Exclusion criteria included: ① Hospital stay < 24 hours; ② No baseline serum magnesium concentration measured within 24 hours of admission; ③ Missing follow-up data; ④ Patients with concurrent pneumonia (to avoid confounding effects of pneumonia-induced sepsis on pulmonary outcomes). Specifically, a total of 359,791 patients were excluded due to missing baseline serum magnesium measurements.The patient screening process is illustrated in Fig 1. All laboratory data, including components for the Sequential Organ Failure Assessment (SOFA) score, were obtained from the first test after admission. SOFA scores were calculated based on available clinical and laboratory data; patients with missing data for one or more SOFA components were recorded as SOFA-N, while those with complete data were recorded as SOFA-Y. The distribution of patients with available and missing SOFA scores across PC-NPS groups and magnesium quartiles is provided in Supplementary S1 Table.

### Data extraction

The following variables were extracted from the MIMIC-IV database:

Demographic characteristics: age, sex;

Blood tests and vital signs: baseline magnesium (Mg²⁺), red blood cells (RBC), red cell distribution width (RDW), hematocrit (Hct), hemoglobin (Hb), platelets (PLT), activated partial thromboplastin time (APTT), prothrombin time (PT), anion gap (AG), bicarbonate (HCO₃⁻), alkaline phosphatase (ALP), alanine aminotransferase (ALT), aspartate aminotransferase (AST), total bilirubin (TBIL), blood urea nitrogen (BUN), creatinine (Cr), calcium (Ca²⁺), chloride (Cl⁻), potassium (K⁺), sodium (Na⁺), phosphate (PO₄³⁻), glucose;

Diagnostic indicators for PC-NPS: hypoxemia, pulmonary edema, acute respiratory failure, pulmonary embolism, pneumothorax. We explicitly defined these composite criteria to standardize the diagnosis within our cohort, thereby ensuring a robust, clinically relevant assessment of the primary endpoint.

Comorbidities: chronic obstructive pulmonary disease (COPD), heart failure (HF), angina pectoris, myocardial infarction (MI), chronic myocardial ischemia, diabetes, chronic kidney disease (CKD), obesity, pneumonia history, HIV infection;Current ICU admission status;History of prior ICU admissions;Follow-up survival status and survival time.

All laboratory data were obtained from the first test after admission. Variables with missing values exceeding 20% were excluded. Variables with missing values below 20% were handled using multiple imputation (R language MICE package).

### Ethical statement

The study adhered to the Declaration of Helsinki. The use of the MIMIC-IV database was approved by the ethics committees of MIT and BIDMC. As the data are publicly available, the requirement for informed consent was waived.

### Endpoints and grouping

The primary endpoint was the occurrence of PC-NPS during the current hospitalization. Secondary endpoints included 90-day all-cause mortality and ICU admission during the current hospitalization.

Patients were first divided into PC-NPS and non-PC-NPS groups based on the primary endpoint to evaluate differences in 90-day all-cause mortality and ICU admission rates. They were then stratified into four groups based on baseline magnesium quartiles to assess differences in PC-NPS incidence, mortality, and ICU admission rates across different magnesium levels.

### Statistical analysis

Before analysis, continuous variables were checked for normality. Normally distributed data are shown as mean ± SD, and t tests were applied for comparisons. For variables not meeting normality, data are summarized as median (IQR) and compared with Mann–Whitney U tests. Categorical variables are presented as number and percentage, with group comparisons based on chi-square tests.

To identify independent risk factors for the incidence of PC-NPS, we performed univariate and multivariate logistic regression analyses. Survival was analyzed using Kaplan–Meier estimates, and log-rank tests were used for group comparisons. For survival analysis regarding 90-day mortality, Kaplan-Meier curves were generated and compared using the log-rank test. Hazard ratios were not calculated as our regression models focused exclusively on the primary endpoint. We also assessed multicollinearity through variance inflation factors; variables with VIF above 5 were excluded. Analyses were performed with R software (version 4.3.1), and statistical significance was defined as a two-tailed p < 0.05.

## Results

### Baseline characteristics

A total of 4,836 septic patients were included in this study. The median age of the patients was 67 years (IQR: 56–78), and 54.28% were male. The incidence of PC-NPS during the current hospitalization was 26.28%, the 90-day mortality rate was 20.72%, and the ICU admission rate was 81.12%. The median magnesium level was 1.9 mg/dL (IQR: 1.6–2.1) (Table 1).

**Table 1:** Baseline characteristics according to quartiles of serum magnesium.

		mg <= 1.6	1.6 < mg<=1.9	1.9 < mg<=2.1	mg > 2.1		
Variables	Total (n = 4836)	Q1 (n = 1257)	Q2 (n = 1729)	Q3 (n = 914)	Q4 (n = 936)	P	statistic
Age, median (Q1–Q3)	67 (56, 78)	66 (54, 76)	67 (56, 79)	68 (55, 80)	69 (58, 79)	< 0.001	21.925
Gender, n (%)						< 0.001	23.349
Male	2625 (54.28)	623 (49.56)	926 (53.56)	539 (58.97)	537 (57.37)		
Female	2211 (45.72)	634 (50.44)	803 (46.44)	375 (41.03)	399 (42.63)		
90-day outcome, n (%)						< 0.001	61.029
Survivor	3834 (79.28)	1064 (84.65)	1381 (79.87)	724 (79.21)	665 (71.05)		
Non-survivor	1002 (20.72)	193 (15.35)	348 (20.13)	190 (20.79)	271 (28.95)		
Mg² ⁺ , Median (Q1,Q3)	1.9 (1.6, 2.1)	1.5 (1.3, 1.6)	1.8 (1.7, 1.9)	2 (2, 2.1)	2.3 (2.2, 2.5)	< 0.001	4498.659
RBC, Median (Q1,Q3)	3.5 (3.01, 4)	3.46 (2.98, 3.99)	3.5 (3.03, 4)	3.55 (3.03, 4.05)	3.5 (2.98, 4)	0.203	4.606
RDW, Median (Q1,Q3)	14.9 (13.8, 16.7)	14.8 (13.7, 16.4)	14.9 (13.7, 16.6)	15 (13.8, 16.7)	15.2 (13.9, 17.2)	< 0.001	29.215
HCT, Median (Q1,Q3)	31.8 (27.6, 36)	31.3 (27.1, 35.7)	31.8 (27.7, 36)	32.3 (27.7, 36.2)	31.95 (27.8, 36)	0.111	6.004
WBC, Median (Q1,Q3)	11.7 (7.8, 16.9)	11.9 (7.4, 17.5)	11.6 (8, 16.8)	11.3 (7.7, 15.6)	11.9 (8.2, 16.9)	0.1	6.25
PLT, Median (Q1,Q3)	185 (127, 261)	167 (117, 240)	188 (129, 262)	199 (131.25, 287.75)	192 (132, 267)	< 0.001	45.533
PT, Median (Q1,Q3)	14.7 (13, 17.7)	15 (13.3, 17.7)	14.7 (13, 17.7)	14.45 (13, 17.4)	14.6 (12.9, 18.1)	0.113	5.964
PTT, Median (Q1,Q3)	31.2 (27.9, 36.1)	31.4 (28.1, 36.2)	31.1 (27.7, 36.1)	31.2 (28.1, 35.5)	31.3 (27.7, 36.9)	0.445	2.671
AG, Median (Q1,Q3)	15 (12, 17)	15 (12, 17)	14 (12, 17)	15 (12, 17)	15 (13, 18)	< 0.001	41.219
HCO₃ ⁻ , Median (Q1,Q3)	22 (20, 25)	21 (19, 24)	23 (20, 25)	23 (20, 26)	23 (20, 26)	< 0.001	110.45
ALP, Median (Q1,Q3)	101 (72, 161)	99 (70, 152)	101 (72, 163)	98 (72, 161.75)	105 (74, 169.25)	0.056	7.57
ALT, Median (Q1,Q3)	26 (15, 53)	25 (14, 48)	25 (15, 51)	25 (15, 52)	30 (17, 60)	< 0.001	21.633
AST, Median (Q1,Q3)	34 (21, 66)	33 (21, 69)	33 (21, 64)	32 (20, 60)	39 (23, 74)	< 0.001	19.656
TBIL, Median (Q1,Q3)	0.6 (0.4, 1.3)	0.7 (0.4, 1.4)	0.6 (0.4, 1.3)	0.5 (0.4, 1.1)	0.7 (0.4, 1.4)	< 0.001	18.549
BUN, Median (Q1,Q3)	23 (14, 38)	20 (13, 29)	21 (13, 33)	23 (15, 38)	38.5 (21, 64.25)	< 0.001	406.383
Cr, Median (Q1,Q3)	1.1 (0.8, 1.9)	1.1 (0.8, 1.7)	1.1 (0.8, 1.6)	1.1 (0.8, 1.8)	1.5 (1, 2.8)	< 0.001	194.455
Ca² ⁺ , Median (Q1,Q3)	8.3 (7.8, 8.8)	8 (7.5, 8.5)	8.4 (7.9, 8.8)	8.5 (8.1, 8.9)	8.5 (8, 9)	< 0.001	273.92
Cl ⁻ , Median (Q1,Q3)	102 (98, 106)	103 (99, 107)	102 (98, 106)	102 (98, 105)	101 (97, 106)	< 0.001	51.417
K ⁺ , Median (Q1,Q3)	4 (3.6, 4.5)	3.8 (3.4, 4.3)	4 (3.6, 4.4)	4.1 (3.7, 4.5)	4.2 (3.8, 4.7)	< 0.001	181.837
Na ⁺ , Median (Q1,Q3)	137 (134, 140)	137 (134, 140)	137 (134, 140)	137 (134, 140)	138 (134, 141)	0.009	11.506
PO₄³ ⁻ , Median (Q1,Q3)	3.2 (2.6, 4)	2.9 (2.4, 3.6)	3.1 (2.5, 3.9)	3.4 (2.7, 4.1)	3.6 (2.9, 4.7)	< 0.001	240.36
Blood glucose, Median (Q1,Q3)	119 (98, 160)	119 (98, 163)	119 (97, 155)	115 (97, 156)	122.5 (99, 170)	0.045	8.031
COPD, n (%)						0.031	8.885
0	4340 (89.74)	1143 (90.93)	1546 (89.42)	832 (91.03)	819 (87.5)		
1	496 (10.26)	114 (9.07)	183 (10.58)	82 (8.97)	117 (12.5)		
HF, n (%)						< 0.001	68.227
0	3337 (69)	938 (74.62)	1219 (70.5)	632 (69.15)	548 (58.55)		
1	1499 (31)	319 (25.38)	510 (29.5)	282 (30.85)	388 (41.45)		
Angina Pectoris, n (%)						0.68	1.51
0	4755 (98.33)	1237 (98.41)	1704 (98.55)	897 (98.14)	917 (97.97)		
1	81 (1.67)	20 (1.59)	25 (1.45)	17 (1.86)	19 (2.03)		
MI, n (%)						< 0.001	21.272
0	4053 (83.81)	1094 (87.03)	1456 (84.21)	755 (82.6)	748 (79.91)		
1	783 (16.19)	163 (12.97)	273 (15.79)	159 (17.4)	188 (20.09)		
CIHD, n (%)						0.224	4.371
0	4716 (97.52)	1234 (98.17)	1687 (97.57)	885 (96.83)	910 (97.22)		
1	120 (2.48)	23 (1.83)	42 (2.43)	29 (3.17)	26 (2.78)		
Diabetes, n (%)						0.013	10.854
0	2897 (59.9)	714 (56.8)	1072 (62)	566 (61.93)	545 (58.23)		
1	1939 (40.1)	543 (43.2)	657 (38)	348 (38.07)	391 (41.77)		
CKD, n (%)						< 0.001	72.391
0	3233 (66.85)	906 (72.08)	1188 (68.71)	619 (67.72)	520 (55.56)		
1	1603 (33.15)	351 (27.92)	541 (31.29)	295 (32.28)	416 (44.44)		
Obesity, n (%)						0.932	0.441
0	3888 (80.4)	1016 (80.83)	1393 (80.57)	732 (80.09)	747 (79.81)		
1	948 (19.6)	241 (19.17)	336 (19.43)	182 (19.91)	189 (20.19)		
Pneumonia history, n (%)						0.527	2.227
0	4008 (82.88)	1042 (82.9)	1447 (83.69)	757 (82.82)	762 (81.41)		
1	828 (17.12)	215 (17.1)	282 (16.31)	157 (17.18)	174 (18.59)		
HIV, n (%)						0.291	3.74
0	4739 (97.99)	1229 (97.77)	1698 (98.21)	890 (97.37)	922 (98.5)		
1	97 (2.01)	28 (2.23)	31 (1.79)	24 (2.63)	14 (1.5)		
ICU history, n (%)						< 0.001	58.585
0	1694 (35.03)	357 (28.4)	647 (37.42)	393 (43)	297 (31.73)		
1	3142 (64.97)	900 (71.6)	1082 (62.58)	521 (57)	639 (68.27)		
ICU, n (%)						< 0.001	41.126
0	913 (18.88)	172 (13.68)	319 (18.45)	206 (22.54)	216 (23.08)		
1	3923 (81.12)	1085 (86.32)	1410 (81.55)	708 (77.46)	720 (76.92)		
PC-NPS, n (%)						< 0.001	26.087
0	3565 (73.72)	936 (74.46)	1308 (75.65)	692 (75.71)	629 (67.2)		
1	1271 (26.28)	321 (25.54)	421 (24.35)	222 (24.29)	307 (32.8)		

PC-NPS, pulmonary complications in non-pulmonary sepsis; Mg, magnesium; RBC, red blood cell count; RDW, red cell distribution width; HCT, hematocrit; WBC, white blood cell count; PLT, Platelet count; PT, Prothrombin time; PTT, Partial thromboPlastin time; AG, anion gaP; HCO₃ ⁻ , bicarbonate; ALP, alkaline PhosPhatase; ALT, alanine aminotransferase; AST, asPartate aminotransferase; TBIL, total bilirubin; BUN, blood urea nitrogen; Cr, creatinine; Ca² ⁺ , calcium; Cl ⁻ , chloride; K ⁺ , Potassium; Na ⁺ , sodium; PO₄³ ⁻ , Phosphate; COPD, chronic obstructive Pulmonary disease; HF, heart failure; MI, myocardial infarction; CIHD, chronic ischemic heart disease; CKD, chronic kidney disease; HIV, human immunodeficiency virus; ICU, intensive care unit.

### Differences in baseline characteristics between the PC-NPS and Non-PC-NPS groups

Compared with the non-PC-NPS group, patients in the PC-NPS group were older (median 68 [IQR: 57–80] vs. 67 [IQR: 55–78] years, P = 0.003), had higher 90-day mortality (32.26% vs. 16.61%, P < 0.001), and although they had a lower current ICU admission rate (74.82% vs. 83.37%, P < 0.001), they had a higher proportion of prior ICU admission history (85.84% vs. 57.53%, P < 0.001). Additionally, the PC-NPS group had significantly higher proportions of comorbidities, including chronic obstructive pulmonary disease (COPD), heart failure (HF), myocardial infarction (MI), chronic kidney disease (CKD), obesity, and a history of pneumonia.

**Fig 2:**
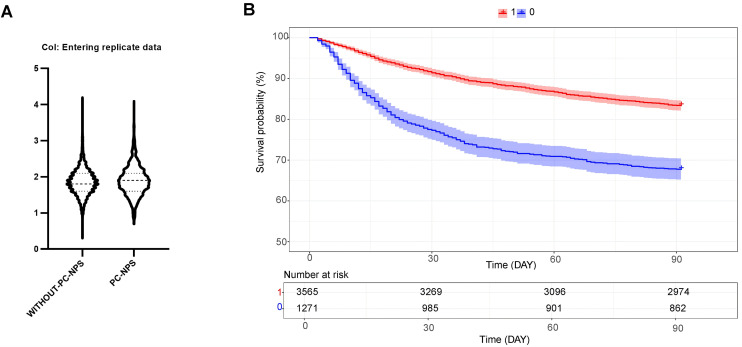
The correlation between serum magnesium ion levels and the occurrence of PC-NPS as well as the prognosis of patients. A: Differences in serum magnesium levels under PC-NPS conditions; B: Patient survival curves under PC-NPS conditions.

In terms of blood indicators, the PC-NPS group exhibited significantly higher levels of magnesium, red cell distribution width (RDW), white blood cell count (WBC), coagulation parameters (APTT, PT), anion gap (AG), liver function markers (alanine aminotransferase [ALT], aspartate aminotransferase [AST], total bilirubin [TBIL]), renal function markers (blood urea nitrogen [BUN], creatinine [Cr]), serum potassium (K⁺), phosphate (PO₄³⁻), and blood glucose. In contrast, platelet count (PLT), bicarbonate (HCO₃⁻), and calcium (Ca²⁺) levels were significantly lower (Table 2, Fig 2A,B).

**Table 2:** Baseline characteristics of patients with and without PC-NPS.

Variables	Total (n = 4836)	Without PC-NPS (n = 3565)	With PC-NPS (n = 1271)	P Value	Statistic
Age, Median (Q1,Q3)	67 (56, 78)	67 (55, 78)	68 (57, 80)	0.003	2136977
Gender, n (%)				0.665	0.187
male	2625 (54.28)	1928 (54.08)	697 (54.84)		
female	2211 (45.72)	1637 (45.92)	574 (45.16)		
90-day outcome, n (%)				< 0.001	138.789
survivor	3834 (79.28)	2973 (83.39)	861 (67.74)		
non-survivor	1002 (20.72)	592 (16.61)	410 (32.26)		
Mg² ⁺ , median (Q1–Q3)	1.9 (1.6, 2.1)	1.8 (1.6, 2.1)	1.9 (1.6, 2.1)	0.008	2152861.5
RBC, median (Q1–Q3)	3.5 (3.01, 4)	3.51 (3.01, 4.01)	3.48 (3.01, 3.98)	0.209	2319199
RDW, median (Q1–Q3)	14.9 (13.8, 16.7)	14.8 (13.7, 16.5)	15.3 (14, 16.9)	< 0.001	2008784
HCT, median (Q1–Q3)	31.8 (27.6, 36)	31.8 (27.5, 36)	31.9 (27.8, 36)	0.787	2254035.5
WBC, median (Q1–Q3)	11.7 (7.8, 16.9)	11.4 (7.7, 16.4)	12.5 (8.2, 18.2)	< 0.001	2050488
PLT, median (Q1–Q3)	185 (127, 261)	189 (130, 263)	176 (116, 251)	< 0.001	2425949.5
PT, median (Q1–Q3)	14.7 (13, 17.7)	14.4 (12.9, 17.3)	15.3 (13.4, 19)	< 0.001	1974210.5
PTT, median (Q1–Q3)	31.2 (27.9, 36.1)	31.1 (27.8, 35.8)	31.5 (28.1, 37.4)	0.034	2175106.5
Anion gaP (AG), median (Q1–Q3)	15 (12, 17)	15 (12, 17)	15 (13, 18)	< 0.001	2116261.5
HCO₃ ⁻ , median (Q1–Q3)	22 (20, 25)	23 (20, 25)	22 (19, 25)	< 0.001	2492747.5
ALP, median (Q1–Q3)	101 (72, 161)	100 (71, 160)	103 (73, 164)	0.19	2209562
ALT, median (Q1–Q3)	26 (15, 53)	26 (15, 51)	27 (16, 60)	0.003	2136852
AST, median (Q1–Q3)	34 (21, 66)	32 (20, 62)	39 (23, 80)	< 0.001	2007414
TBIL, median (Q1–Q3)	0.6 (0.4, 1.3)	0.6 (0.4, 1.2)	0.8 (0.4, 1.6)	< 0.001	2016008
BUN, median (Q1–Q3)	23 (14, 38)	22 (14, 36)	27 (17, 44)	< 0.001	1897217
Cr, median (Q1–Q3)	1.1 (0.8, 1.9)	1.1 (0.8, 1.8)	1.3 (0.8, 2.1)	< 0.001	2038109
Ca² ⁺ , median (Q1–Q3)	8.3 (7.8, 8.8)	8.4 (7.9, 8.8)	8.2 (7.7, 8.7)	< 0.001	2510970.5
Cl ⁻ , median (Q1–Q3)	102 (98, 106)	102 (98, 106)	102 (98, 106)	0.855	2257732.5
K ⁺ , median (Q1–Q3)	4 (3.6, 4.5)	4 (3.6, 4.4)	4.1 (3.7, 4.6)	< 0.001	2046769
Na ⁺ , median (Q1–Q3)	137 (134, 140)	137 (134, 140)	137 (134, 140)	0.134	2201635
PO₄³ ⁻ , median (Q1–Q3)	3.2 (2.6, 4)	3.1 (2.5, 3.9)	3.4 (2.7, 4.4)	< 0.001	1928007
Blood glucose, median (Q1–Q3)	119 (98, 160)	117 (97, 156)	125 (99, 170)	< 0.001	2108151
COPD, n (%)				< 0.001	67.223
0	4340 (89.74)	3276 (91.89)	1064 (83.71)		
1	496 (10.26)	289 (8.11)	207 (16.29)		
HF, n (%)				< 0.001	85.022
0	3337 (69)	2591 (72.68)	746 (58.69)		
1	1499 (31)	974 (27.32)	525 (41.31)		
Angina Pectoris, n (%)				0.335	0.93
0	4755 (98.33)	3501 (98.2)	1254 (98.66)		
1	81 (1.67)	64 (1.8)	17 (1.34)		
MI, n (%)				< 0.001	24.412
0	4053 (83.81)	3044 (85.39)	1009 (79.39)		
1	783 (16.19)	521 (14.61)	262 (20.61)		
CIHD, n (%)				0.534	0.387
0	4716 (97.52)	3480 (97.62)	1236 (97.25)		
1	120 (2.48)	85 (2.38)	35 (2.75)		
Diabetes, n (%)				0.784	0.075
0	2897 (59.9)	2131 (59.78)	766 (60.27)		
1	1939 (40.1)	1434 (40.22)	505 (39.73)		
CKD, n (%)				0.05	3.83
0	3233 (66.85)	2412 (67.66)	821 (64.59)		
1	1603 (33.15)	1153 (32.34)	450 (35.41)		
Obesity, n (%)				< 0.001	11.577
0	3888 (80.4)	2908 (81.57)	980 (77.1)		
1	948 (19.6)	657 (18.43)	291 (22.9)		
Pneumonia history, n (%)				< 0.001	44.461
0	4008 (82.88)	3032 (85.05)	976 (76.79)		
1	828 (17.12)	533 (14.95)	295 (23.21)		
HIV, n (%)				0.103	2.656
0	4739 (97.99)	3486 (97.78)	1253 (98.58)		
1	97 (2.01)	79 (2.22)	18 (1.42)		
ICU history, n (%)				< 0.001	328.625
0	1694 (35.03)	1514 (42.47)	180 (14.16)		
1	3142 (64.97)	2051 (57.53)	1091 (85.84)		
ICU, n (%)				< 0.001	44.095
0	913 (18.88)	593 (16.63)	320 (25.18)		
1	3923 (81.12)	2972 (83.37)	951 (74.82)		
Variables	Total (n = 4836)	Without PC-NPS(n = 3565)	With PC-NPS (n = 1271)	P	statistic
Age, Median (Q1,Q3)	67 (56, 78)	67 (55, 78)	68 (57, 80)	0.003	2136977

PC-NPS, pulmonary complications in non-pulmonary sepsis; RBC, red blood cell count; RDW, red cell distribution width; HCT, hematocrit; WBC, white blood cell count; PLT, Platelet count; PT, Prothrombin time; PTT, Partial thromboPlastin time; AG, anion gaP; HCO₃ ⁻ , bicarbonate; ALP, alkaline PhosPhatase; ALT, alanine aminotransferase; AST, asPartate aminotransferase; TBIL, total bilirubin; BUN, blood urea nitrogen; Cr, creatinine; Ca² ⁺ , calcium; Cl ⁻ , chloride; K ⁺ , Potassium; Na ⁺ , sodium; PO₄³ ⁻ , Phosphate; COPD, chronic obstructive Pulmonary disease; HF, heart failure; MI, myocardial infarction; CIHD, chronic ischemic heart disease; CKD, chronic kidney disease; HIV, human immunodeficiency virus; ICU, intensive care unit

### Baseline characteristics stratified by magnesium quartiles

Patients were divided into four groups based on magnesium quartiles: Q1 (<1.6 mg/dL), Q2 (1.6–1.9 mg/dL), Q3 (1.9–2.1 mg/dL), and Q4 (>2.1 mg/dL). From Q1 to Q4, patient age gradually increased(median: 66 [IQR: 54–76], 67 [IQR: 56–79], 68 [IQR: 55–80], and 69 [IQR: 58–79] years, P < 0.001). Among comorbidities, the prevalence of HF, MI, and CKD showed an increasing trend (all P < 0.001). COPD was slightly lower only in Q3 but overall increased with rising magnesium concentrations (P = 0.031).

Regarding laboratory parameters, RDW increased from Q1 to Q4 (P < 0.001). Platelet (PLT) levels generally trended upward, peaking in Q3 (P < 0.001). Bicarbonate (HCO₃⁻) levels increased from Q1 to Q3 (P < 0.001). Among liver and kidney function markers, ALT and AST were significantly elevated in Q4 (both P < 0.001). BUN and Cr showed a marked increasing trend, particularly in Q4 (both P < 0.001). For electrolyte indicators, Ca² ⁺ , K ⁺ , and PO₄³ ⁻ increased across quartiles (all P < 0.001), while Na ⁺ was significantly higher in Q4 (P = 0.009). Conversely, Cl⁻ decreased across quartiles (P < 0.001).

**Fig 3:**
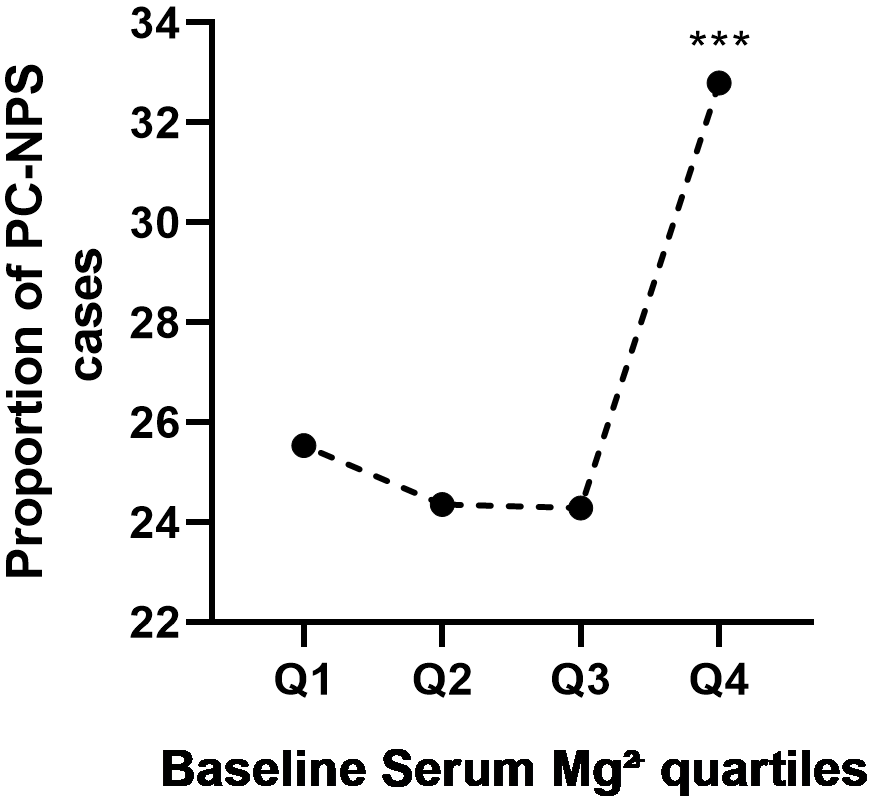
Relationship between serum magnesium level quartile groups and the incidence of PC-NPS. Relationship between baseline serum magnesium quartiles (Q1–Q4) and incidence of pulmonary complications in non-pulmonary sepsis (PC-NPS). Q1–Q4 represent magnesium concentrations <1.6, 1.6–1.9, 1.9–2.1, and >2.1 mg/dL, respectively. Data shown as proportion (%) of patients within each quartile developing PC-NPS.

Additionally, the current ICU admission rate significantly decreased from Q1 to Q4 (86.32% → 81.55% → 77.46% → 76.92%, P < 0.001), while mortality (15.35% → 20.13% → 20.79% → 28.95%, P < 0.001) and PC-NPS incidence (25.54% → 24.35% → 24.29% → 32.8%) significantly increased (all P < 0.001) (Table 1, Fig 3).

### Survival analysis

**Fig 4:**
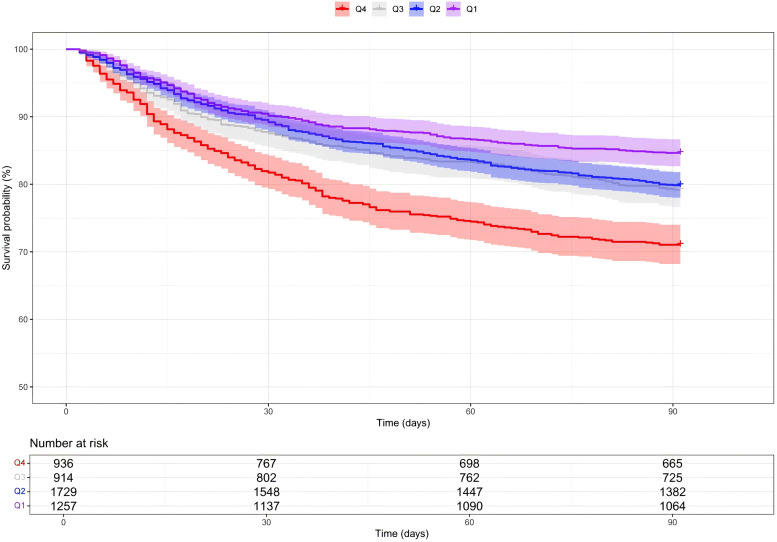
Comparison of survival curves across magnesium ion quartile distributions. Kaplan-Meier survival curves of 90-day mortality stratified by baseline serum magnesium quartiles (Q1–Q4). Shaded areas indicate 95% confidence intervals. Numbers at risk are shown below the x-axis.

Regarding clinical outcomes, Kaplan-Meier survival curves demonstrated a significant separation between the PC-NPS and non-PC-NPS groups, with the PC-NPS group exhibiting a higher cumulative 90-day mortality (log-rank P < 0.001; Fig 2B). Furthermore, when stratified by serum magnesium quartiles, the highest quartile (Q4, > 2.1 mg/dL) showed the poorest survival, with the highest cumulative 90-day mortality (log-rank P < 0.001; Fig 4).

To identify risk factors for the development of PC-NPS, univariate logistic regression analysis was performed (Table 3). The results demonstrated that elevated serum magnesium levels were significantly associated with an increased risk of PC-NPS (Odds Ratio [OR] = 1.322, 95% CI:1.117–1.566, P = 0.001). Additionally, age, history of ICU admission, and comorbidities such as COPD, asthma, and heart failure were also identified as significant risk factors for the occurrence of PC-NPS (P < 0.05).

**Table 3:** Results of univariate logistic regression analysis for PC-NPS.

Variable	Odds Ratio (95% CI)	P Value
Age	1.005 (1.002-1.009)	< 0.001
Gender	0.97 (0.853-1.103)	0.642
Magnesium	1.322 (1.117-1.566)	0.001
RBC	0.936 (0.856-1.023)	0.147
RDW	1.072 (1.045-1.099)	< 0.001
Hematocrit	1.001 (0.991-1.012)	0.824
WBC	1.015 (1.009-1.023)	< 0.001
PLT	0.999 (0.998-0.999)	< 0.001
PT	1.014 (1.008-1.019)	< 0.001
PTT	1.004 (1-1.007)	0.035
AG	1.036 (1.02-1.053)	< 0.001
HCO3⁻	0.963 (0.949-0.977)	< 0.001
ALP	1 (0.999-1)	0.605
ALT	1 (1-1.001)	< 0.001
AST	1 (1–1)	< 0.001
TBIL	1.03 (1.013-1.046)	< 0.001
BUN	1.009 (1.007-1.012)	< 0.001
Cr	1.029 (0.996-1.062)	0.082
Ca²⁺	0.815 (0.754-0.88)	< 0.001
Cl⁻	1.002 (0.992-1.012)	0.723
K⁺	1.234 (1.137-1.338)	< 0.001
Na⁺	1.009 (0.997-1.022)	0.133
PO₄³⁻	1.19 (1.134-1.247)	< 0.001
Glu	1.001 (1-1.002)	< 0.001
COPD	2.205 (1.82-2.669)	< 0.001
Asthma	1.946 (1.306-2.874)	< 0.001
HF	1.872 (1.637-2.14)	< 0.001
Angina Pectoris	0.742 (0.42-1.241)	0.277
MI	1.517 (1.286-1.787)	< 0.001
CIHD	1.159 (0.769-1.712)	0.468
Diabetes	0.98 (0.859-1.116)	0.759
Chronic kidney disease	1.147 (1.002-1.311)	0.047
Obesity	1.314 (1.124-1.535)	< 0.001
Pneumonia history	1.719 (1.465-2.015)	< 0.001
HIV	0.634 (0.367-1.037)	0.083
ICU history	4.474 (3.78-5.323)	< 0.001
ICU	0.593 (0.508-0.692)	< 0.001

RBC, red blood cell count; RDW, red cell distribution width; Hematocrit, hematocrit; WBC, white blood cell count; PLT, platelet count; PT, prothrombin time; PTT, partial thromboplastin time; AG, anion gap; HCO₃ ⁻ , bicarbonate; ALP, alkaline phosphatase; ALT, alanine aminotransferase; AST, aspartate aminotransferase; TBIL, total bilirubin; BUN, blood urea nitrogen; Cr, creatinine; Ca² ⁺ , calcium; Cl ⁻ , chloride; K ⁺ , potassium; Na ⁺ , sodium; PO₄³ ⁻ , phosphate; Glu, glucose; COPD, chronic obstructive pulmonary disease; Asthma, asthma; HF, heart failure; Angina Pectoris, angina pectoris; MI, myocardial infarction; CIHD, chronic ischemic heart disease; Diabetes, diabetes; Chronic kidney disease, chronic kidney disease; Obesity, obesity; Pneumonia history, pneumonia history; HIV, human immunodeficiency virus; ICU history, intensive care unit history; ICU, intensive care unit.

### Primary outcome

Multivariate logistic regression analysis, using the backward stepwise method, revealed that an increase in magnesium concentration was significantly associated with a higher risk of PC-NPS significantly rising (OR=1.252, 95% CI: 1.04–1.506, P = 0.017) (Table 4). Restricted cubic spline analysis uncovered a “U-shaped” nonlinear relationship between serum magnesium concentration and risk (OR), which has two critical inflection points (1.26 mg/dL and 1.91 mg/dL).

**Table 4:** Results of multivariate logistic regression analysis for PC-NPS.

Variable	Odds Ratio	95% CI	P Value
Serum magnesium (Mg²⁺)	1.252	1.04-1.506	0.0173
WBC	1.011	1.004-1.018	0.0021
PLT	0.999	0.998-0.999	0.0002
HCO3	0.985	0.968-1.001	0.0657
Serum calcium (Ca²⁺)	0.899	0.825-0.98	0.0158
Serum Potassium (K⁺)	1.114	1.014-1.223	0.0244
Serum Phosphate (PO₄³⁻)	1.066	1.014-1.126	0.0179
COPD	1.847	1.498-2.274	<0.0001
HF	1.397	1.195-1.631	<0.0001
CKD	0.803	0.683-0.942	0.0072
Pneumonia history	1.265	1.059-1.508	0.0092
ICU history	3.525	2.951-4.23	<0.0001
ICU	0.607	0.514-0.717	<0.0001

WBC, white blood cell; PLT, Platelet; HCO₃ ⁻ , bicarbonate; Ca² ⁺ , calcium; K ⁺ , Potassium; PO₄³ ⁻ , Phosphate; COPD, chronic obstructive Pulmonary disease; HF, heart failure; CKD, chronic kidney disease; ICU, intensive care unit.

**Fig 5:**
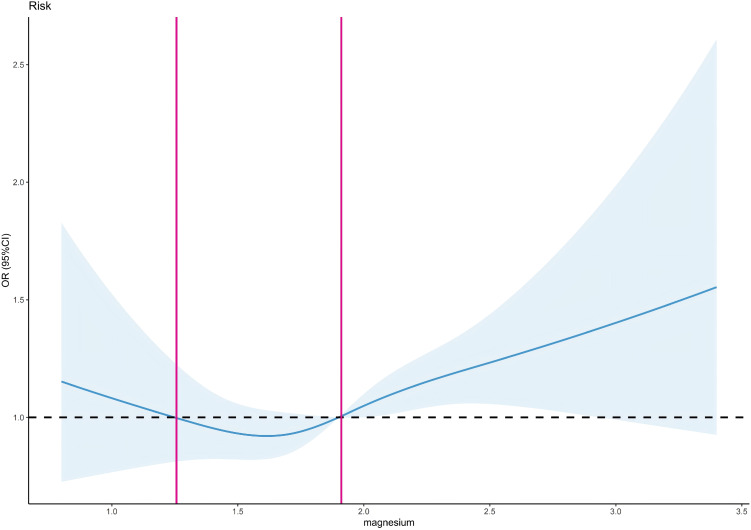
Restricted cubic spline analysis of magnesium ions. Restricted cubic spline analysis showing U-shaped relationship between baseline serum magnesium concentration (mg/dL) and odds ratio (OR) for PC-NPS. Shaded area represents 95% confidence interval; dashed line indicates OR=1; vertical lines denote inflection points at 1.26 and 1.91 mg/dL.

In the low magnesium risk zone (<1.26 mg/dL), with the decreasing of magnesium concentration, the OR value increase rapidly. Noteworthy, the confidence interval crossed 1 remind that the trend of increased risk was not significant when magnesium concentration was < 1.5 mg/dL. In the safety window interval (1.26–1.91 mg/dL), the OR value fluctuated around 1.0, finding the lowest risk within this range. In the high magnesium risk zone (>1.91 mg/dL), the OR value gradually increased with rising magnesium concentration. With the confidence interval did not cross 1, demonstrating a significant increase in risk when magnesium concentration exceeded 1.91 mg/dL (Fig 5, Table 5).

**Table 5:** Parameters of restricted cubic spline analysis for the association between serum magnesium and PC-NPS.

Variables	Knots	Reference 1(OR=1)	Reference 2(OR=1)	P-overall	P-nonlinear
Serum Magnesium	4	1.257	1.911	0.0375	0.2032

RCS:restricted cubic spline; OR: odds ratio.

## Discussion

This study is the first to reveal a nonlinear U-shaped dose-response relationship between serum magnesium levels and PC-NPS in a large sepsis cohort. We identified a potential safety range for serum magnesium in clinical management, 1.26–1.91 mg/dL. Beyond this range, particularly in the highest quartile (Q4, > 2.1 mg/dL), the incidence of PC-NPS increased from 25.54% (Q1) to 32.8%, and 90-day mortality rose from 15.35% to 28.95%. Our findings underscore the importance of magnesium level control in sepsis management, which has emphasized that the serum magnesium concentration remain inferior to 1.91 mg/dL and also proposed to maintain it within the safe range of 1.26–1.91 mg/dL. Notably, the significantly lower ICU admission rate in the hypermagnesemia group (76.92% vs. 86.32%) may indicate rapid deterioration or even death before ICU transfer in some patients. Overall, these results warrant enhanced early warning and dynamic monitoring for patients with magnesium dysregulation, particularly hypermagnesemia.

The pathogenesis of hypermagnesemia may involve multiple pathophysiological processes. Previous studies reported anti-inflammatory effects of magnesium via inhibition of the HMGB1/TLR4/NF-κB pathway; however, other studies have observed elevated levels of pro-inflammatory cytokines (e.g., IL-6, TNF-α) in hypermagnesemia^8^. Regard as coagulation abnormalities, we observed a paradoxical phenomenon: prolonged APTT and PT alongside thrombocytopenia in the hypermagnesemia group. Which might reflect a direct inhibition of coagulation factor activity by magnesium ions (e.g., competitive antagonism of calcium-dependent thrombin generation) combined with endothelial injury effects. Similarly, the high concentration of magnesium may expedite the mitochondrial dysfunction by modulating P2X7 receptor signaling. Hypermagnesemia promots ferroptosis-related lipid peroxidation [9], which could be a key mechanism in alveolar epithelial damage during PC-NPS. Therefore, we had found elevated phosphate levels in the hypermagnesemia group, which may promote mitochondrial membrane permeability transition pore opening, aggravating ferroptosis-related injury [9], providing new directions for future mechanistic research.

Our results offer new perspectives on magnesium management in septic patients. Consistent with previous studies showing hypomagnesemia increases sepsis mortality risk, the lowest magnesium quartile (Q1) in our study had a mortality rate of 15.35%. However, the hypermagnesemia group (Q4) showed a further increase to 28.95%, suggesting a “bidirectional” association between magnesium concentration and sepsis prognosis. Notably, while hypomagnesemia typically worsens prognosis by exacerbating microcirculatory dysfunction and lactic acidosis, we found significantly elevated anion gap and lactate levels in the hypermagnesemia group, indicating that metabolic acidosis may serve as a critical mediator of mortality risk regardless of magnesium status [10, 11, 12, 13, 14]. Therefore, dynamic monitoring of serum magnesium levels shows potential practical value for predicting sepsis-associated PC-NPS. For patients whose magnesium levels fall outside the safety window, strict monitoring and prevention of metabolic acidosis are essential.

There are still several limitations need to careful consideration. The retrospective design can’t fully eliminate the residual confounding factors, which influenced the causal relationship between serum magnesium levels and outcomes, such as the dose and timing of magnesium supplementation. Similarly, the MIMIC-IV database lacks the localized microenvironment indicators of patients (e.g., bronchoalveolar lavage fluid), making it difficult to distinguish the distribution of magnesium in serum or lung tissue. Moreover, the study did not dynamically assess the impact of magnesium fluctuations on prognosis. Also, the magnesium homeostasis during sepsis may hold greater clinical significance than single measurements. Additionally, as a common cause of sepsis, pneumonia patients had been excluded to reduce the confouding bias, the underestimate of true correlation between magnesium levels and PC-NPS is still remaind. Furthermore, our study relied on ICD-9 and ICD-10 administrative codes to identify patients with sepsis from the MIMIC-IV database. Previous validation studies have demonstrated that while administrative claims data possess adequate specificity, their sensitivity for identifying true sepsis cases is relatively low and variable compared to clinical criteria [19, 20]. Consequently, a proportion of patients who clinically met the Sepsis-3 criteria might not have been captured in our cohort. This reliance on billing codes may introduce selection bias and could affect the generalizability of our findings to clinical settings that utilize real-time physiological and laboratory criteria for sepsis identification.

Future research should: 1) Conduct prospective cohort studies to validate the magnesium safety window, combined with multi-omics approaches to elucidate hypermagnesemia-lactate metabolism interactions; 2) Use animal models to investigate magnesium’s regulatory mechanisms on alveolar epithelial ferroptosis and pyroptosis, particularly focusing on P2X7 receptor signaling pathways [9, 10]; 3) Develop risk models based on dynamic magnesium changes for prognostic stratification; and 4) Evaluate how different magnesium concentrations affect responses to anticoagulant therapy to inform personalized coagulation management.

In summary, our study shows a U-shaped association between serum magnesium levels and sepsis-associated PC-NPS risk/mortality: the 1.26–1.91 mg/dL safety window for preventing sepsis-associated pulmonary complications. These findings suggest strengthening dynamic monitoring of magnesium levels in septic patients.It warns us to avoid blindly supplementation that leads to excessive concentrations. While closely monitoring early organ dysfunction and fluid-electrolyte/acid-base balance changes in patients with dysmagnesemia. Future research should explore how magnesium concentration thresholds influence immunometabolic reprogramming. Such work could offer fresh insights into precision medicine for sepsis and help shape more individualized treatment strategies.

## Conclusion

In this current study, we identified a U-shaped, nonlinear association between serum magnesium levels and the risk of PC-NPS of the large retrospective cohort of septic patients which from the MIMIC-IV database. A safety window of 1.26–1.91 mg/dL was associated with the lowest risk of PC-NPS and mortality by the adverse outcomes of both hypomagnesemia and hypermagnesemia. These findings highlight the importance of dynamic monitoring of magnesium levels during sepsis, while revealing that avoiding excessive supplementation and maintaining serum concentrations within the optimal range may reduce the risk of sepsis-associated pulmonary complications. Future prospective studies and mechanistic investigations are warranted to validate these thresholds, to further investigate the immunometabolic mechanism through which magnesium regulates PC-NPS.

## Supporting information

S1 Data Codes. (ZIP)

S1 Table Availability of SOFA scores in the cohort. (DOCX)
