# Correction: The safety window of blood magnesium in pulmonary complications of non-pulmonary sepsis: A U-shaped risk and prognostic analysis based on MIMIC-IV

**DOI:** 10.1371/journal.pone.0361591

**Published:** 2026-10-27

**Authors:** Taotao Peng, Yu Li, Yukun Ren, Mi Yang, Zonghong Long, Dukun Zuo, Lu Huang, Huawei Liu, Zhenxin Duan, Hong Li

The Funding statement for this article is incorrect. The correct Funding statement is as follows: This research was financially supported by the Institute of Science and Technology, National Health Commission of China — ‘Clinical Study on Precision Anesthesia Depth Monitoring and Patient Prognosis’ (Grant No. 2024KYS01803 to Zonghong Long).
