# Supplementary material for: The safety window of blood magnesium in pulmonary complications of non-pulmonary sepsis: A U-shaped risk and prognostic analysis based on MIMIC-IV
Source: PLoS One. 2026 Jun 15;21(6):e0351216. doi: 10.1371/journal.pone.0351216 (PMC13268139; doi:10.1371/journal.pone.0351216)
Supplement: S1 Table — (DOCX) [file pone.0351216.s002.docx]

Supplementary Table 1: Availability of SOFA scores in the cohort.

| Group | Total patients | SOFA-Y | SOFA-N |
| --- | --- | --- | --- |
| PC-NPS-Y | 1271 | \| 345 \| \| --- \| | \| 926 \| \| --- \| |
| PC-NPS-N | 3565 | 2 | 3563 |

| Magnesium quartile | Total patients | SOFA-Y | SOFA-N |
| --- | --- | --- | --- |
| Q1 | 1257 | 90 | 1167 |
| Q2 | 1729 | 108 | 1621 |
| Q3 | 914 | 62 | 852 |
| Q4 | 936 | 87 | 849 |

SOFA-Y indicates patients with complete SOFA scores, SOFA-N indicates patients with missing SOFA components.

Supplementary Note regarding missing SOFA scores: Due to the retrospective nature of the MIMIC-IV database, the absence of complete SOFA scores in the excluded cohort (SOFA-N) was primarily driven by the lack of specific laboratory parameters rather than clinical missingness. In routine clinical practice for non-mechanically ventilated or less severe sepsis patients, invasive tests such as arterial blood gas analysis (required for the respiratory component, PaO2/FiO2) and daily liver function tests (required for the hepatic component, bilirubin) are not continuously mandated. Consequently, the missingness of SOFA scores in this subset largely reflects a lower clinical suspicion or indication for these specific tests at baseline, rather than random data loss.
